# ‘*They are with me on this journey*’, the importance of participant-researcher relationships on attendance at follow-up in a behavioural weight management trial: a qualitative study of participant perspectives

**DOI:** 10.1186/s13063-026-09818-w

**Published:** 2026-06-06

**Authors:** Claire Torrens, Catriona O’Dolan, Alice MacLean, Gozde Ozakinci, Barbara Farquharson

**Affiliations:** 1 University of Stirling, Stirling, UK; 2 Glasgow Caledonian University, Glasgow, UK

**Keywords:** Retention, Participant-researcher relationships, Weight management, Obesity

## Abstract

**Background:**

Retaining people for the duration of a trial or study can be challenging. This has been a particular difficulty for trials of behavioural weight management programmes. Poor retention can detrimentally impact data quality, validity, reliability, and knowledge translation. A number of behavioural strategies (for example reminders or monetary rewards) have been implemented to improve retention at follow-up in trials. However, research in this area is limited, the evidence is uncertain, and has not considered wider strategies such as style of delivery or the role of participant-researcher relationships within trial processes. The aim of this qualitative study was to explore participant views and experiences of follow-up weight assessments.

**Methods:**

Participants (*n* = 390) were randomised to protocolised weight assessments, scheduled at 3 and 6 months. These were implemented in an embedded study within a trial of a 12-month text-based behavioural weight management programme. Participants received a task-oriented approach focused on completing weight verification and the case report form or a relational approach focused on building the participant relationship. Assessments were delivered by researchers at three recruiting centres (Belfast, Bristol, and Glasgow). Framework method was used to analyse transcripts of semi-structured interviews with participants.

**Results:**

Fifty-four trial participants took part in interviews. Three overarching themes were constructed from the narratives: the value of attending follow-up weight assessments, mode of weight assessment delivery, and contextual and practical factors influencing attendance at weight assessments. The themes provided novel insights into attendance at follow-up outcome assessments in a weight management trial including the emotive nature of attending weight assessments, related to weight stigma. Little distinguished the two groups. Participants spoke of positive relationships with researchers, benefitted from the researcher role within assessments, and desired in-person human contact, each considered by participants to enhance attendance.

**Conclusion:**

Participant-researcher relationships have an important role in retention. How researchers approach trial processes, such as outcome assessments, could improve attendance within weight management trials primarily by supporting motivation through mechanisms of change such as a sense of responsibility or accountability. Removal of connection through digital-only interventions, with limited in-person follow-up, has potential implications for retention.

**SWAT registration:**

Northern Ireland Hub for Trials Methodology Research SWAT 147, 1/04/2020.

**Supplementary Information:**

The online version contains supplementary material available at 10.1186/s13063-026-09818-w.

## Background

Retention within trials and studies of behavioural weight management interventions (BWMI) can range widely and can be dependent on a range of factors including the length of study and sociodemographic factors such as gender [1]. Poor retention can be costly, impacting the quality, reliability, and validity of data making interpretation and knowledge translation more difficult [2, 3]. Retention strategies, including specific behaviour change techniques, such as prompts and cues, reminders, and incentives (e.g. monetary rewards) have been investigated [3–5]. However, evidence of effectiveness of these strategies is uncertain or weak, largely concerned with improving questionnaire responses and strategies can be costly [3, 4, 6]. Despite retention in trials being a top research priority [7], key questions related to retention, such as ‘what motivates a participant’s decision to complete a clinical trial?’ and ‘how does a participant’s ongoing experience of the trial affect retention?’, remain largely unanswered [8].

In studies of weight management, the ability to develop bonds through trusting relationships [9] and providing space for weight conversations [10] during in-person contacts have been shown to carry importance both for intervention effectiveness and retention [6, 11–14]. Digital interventions, such as those investigated in weight management trials, can reach large numbers, as well as more disadvantaged groups often underrepresented in face-to-face intervention trials [15–17]. However, they can also vary in level of interaction between participants and research teams [17]. It is not known how or if important factors, such as developing rapport or trust, translate to trials of digital interventions. Regardless of in-person or digital delivery, the success of BWMIs depends upon ongoing engagement [18]. Understanding the factors that help to retain different participants with consideration of mode of delivery [19] could support the development of effective retention strategies. Exploring retention strategies, such as the style of delivery and utilising the participant-researcher relationship, beyond the limited scope of the existing evidence base is therefore timely.

This study aimed to explore participants' views and experiences of protocolised follow-up weight assessments during participation in a BWMI trial.

## Methods

### Design

This qualitative study formed part of an embedded study within a trial (SWAT) exploring the influence of participant-researcher relationships on attendance at follow-up assessments. The SWAT was registered on the Northern Ireland Hub for Trials Methodology Research repository [20]. This study is reported according to the Consolidated Criteria for Reporting Qualitative Research (COREQ) [21] (Additional File 1).

**Fig. 1:**
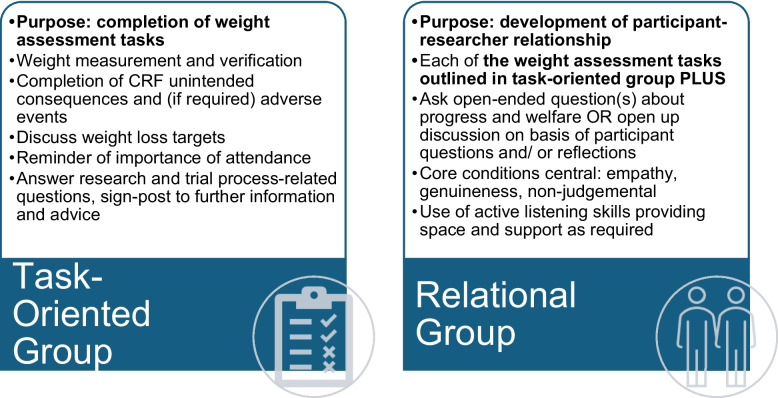
Overview of protocolised SWAT groups

The host trial, Game of Stones [22, 23] (ISRCTN 91974895), was conducted across three UK centres (Belfast, Bristol, and Glasgow) and randomised 585 men to one of three groups, texts with financial incentives (*n* = 196), texts alone (*n* = 194), and a waiting list (*n* = 195). A second randomisation allocated intervention participants (*n* = 390) to two protocolised weight assessments (these are referred to as SWAT groups throughout) delivered by researchers at follow-up at 3 and 6 months (see Additional file 2). The task-oriented group focused on completing the weight assessment tasks. The relational group focused on building a relationship with the participant (see Fig. 1). A qualitative design supported an in-depth exploration of participants’ views and experiences of follow-up assessments to better understand retention, in this case follow-up attendance, and how participant-researcher relationships influence retention within a trial.

### Participants

Participants allocated to the two SWAT groups and who still remained in the trial at 12 months (*n* = 274) were eligible to take part in interviews. Participants were purposively selected to provide representation based on trial centre, the intervention groups (texts alone and texts and financial incentives) in the host trial, the SWAT groups (relational and task-oriented), and diverse health and wellbeing characteristics:

- Self-reported mental health condition (MH)
- Multiple long-term conditions (MLTC)
- Deprivation status (less/more deprived)

Participants were mainly identified through searches of a data management system. Researchers at each centre in the host trial also identified men they considered met the criteria and had the potential to provide useful, in-depth information relevant to the host trial and SWAT objectives.

The sample size for this qualitative study was determined by the host trial [23]. The sample was reviewed throughout guided by information power [24] therefore taking into account the adequacy and relevance of the information that the sample holds in order to answer the research questions. This was judged according to alignment with the aim of the study, the characteristics of the sample, theories of change related to retention, the quality of the dialogue, and the analysis strategy. The wider Game of Stones project management team (PH, KT, KH, SD, CG) reviewed transcripts and the sampling frame throughout data collection to ensure the sample met the needs of the host trial.

Consent to participate in qualitative interviews was provided by participants during their baseline assessment in the host trial and reviewed at the 12-month outcome assessment. If selected to take part, participants were provided with a participant information leaflet (Additional file 3) and contacted by researchers conducting interviews (CT, COD, KM) after their 12-month outcome assessment.

### Data collection

Semi-structured interviews were conducted with individual participants according to their preferred method (telephone, Zoom, or Microsoft Teams) and were audio-recorded. Researchers followed a topic guide including prompts. This incorporated questions specific to the SWAT as well as the host trial (Additional file 4). The questions relevant to the SWAT focused upon participant views and experiences of attending the 3- and 6-month follow-up weight assessments including perceptions about in-person weight assessments.

### Positionality statement

Interviews were conducted by three female researchers, CT (MSc Health Psychology, PhD researcher in health sciences and Research Fellow leading the qualitative sub-study, with experience of behaviour change and weight management), COD (PhD, Trial Manager with behaviour change and qualitative experience), and KM (MSc Public Health, Research Assistant with expertise in food insecurity and qualitative research). CT and KM assessed outcomes and were the main contacts for participants in Glasgow. They did not carry out interviews with participants from this centre. COD was a trial manager and had limited contact with any of the men taking part in the study so conducted interviews in the Glasgow area. Therefore, researchers carrying out the interviews had no close relationships with the participants they interviewed. Analysis was conducted by CT, COD, and AM (PhD, Research Fellow overseeing the qualitative aspects of the host trial process evaluation with experience of behaviour change intervention development and evaluation).

### Analysis

Interviews were transcribed verbatim and de-identified, removing any potentially identifiable references to people or places, by members of the trial team (COD, LM) and uploaded for analysis into QSR NVivo (v15.1) software. Analysis of the interviews was guided by the seven stages of framework method [25]: 1. transcription; 2. familiarisation; 3. coding; 4. identifying and developing a thematic framework; 5. applying the analytical framework—indexing; 6. charting data into the framework matrix; 7. interpretating the data (Additional file 5). To standardise indexing and charting, a code book was developed (Additional file 6). Any quotations were also anonymised and/or made non-attributable when reporting qualitative findings. Only the data derived from the SWAT specific questions in the topic guide and relevant for retention were analysed for this study. To minimise the risk of bias, analysis and interpretation of the qualitative data was completed prior to analysis of the quantitative results of the SWAT.

## Results

Fifty-four men (aged 24–81 years) participated in qualitative interviews between September 2022 and May 2023 (average: 40 min; range: 13–81 min). The majority of men (89%) were white and had attended both the 3- and 6-month assessments (90%) with low non-attendance (9%), i.e. missed attending either the 3- or 6-month follow-up weight assessment (Table 1).

**Table 1:** Participant characteristics

Characteristic	Relational (*n* = 31)	Task-oriented (*n* = 23)	Total (*n* = 54)
Trial centre			
Belfast	8	9	17
Bristol	11	5	16
Glasgow	12	9	21
Ethnicity			
White	28	20	48
Black, African, Caribbean	0	1	1
Other ethnic group	0	1	1
Prefer not to say	1	0	1
Not reported	2	1	3
Deprivation category			
IMD 4–5 (less deprived)	18	10	28
IMD 1–3 (more deprived)	13	13	26
Self-reported health and wellbeing			
Mental health condition	6	5	11
MLTC (not including mental health condition)	8	5	13
Mental health condition and MLTC	3	2	5
No health conditions	14	11	25
Attendance at follow-up weight assessments			
Attended one weight assessment	2	3	5
Attended two weight assessments	29	20	49
Host trial intervention group			
Texts alone	16	8	24
Texts and financial incentives	15	15	30

*IMD* Index Multiple Deprivation, *MLTC* multiple long-term conditions

### Themes

**Fig. 2:**
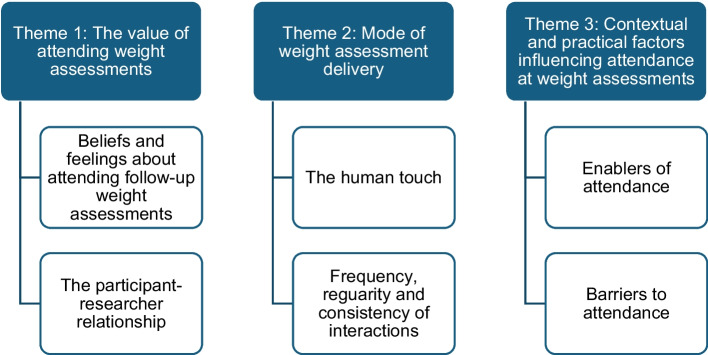
Overview of themes and sub-themes

Three overarching themes, each with two sub-themes (see Fig. 2), were constructed from the interview data: theme 1: the value of attending follow-up weight assessments, theme 2: mode of weight assessment delivery, and theme 3: contextual and practical factors influencing attendance at weight assessments. Similarities between SWAT groups followed by differences under each theme are presented with illustrative quotes. In terms of the outlined SWAT characteristics, there were no patterns found in relation to deprivation, mental health, or multiple long-term conditions. However, physical health was connected with retention and is described under the sub-theme ‘barriers to attendance’.

#### Theme 1: the value of attending follow-up weight assessments

##### Sub-theme: beliefs and feelings about attending follow-up weight assessments

Many participants in both the SWAT groups talked about having positive experiences in relation to their weight assessments, looking forward to, and enjoying them.

I’ve got to say I quite enjoyed it actually. The 3 month one I had some trepidations over because it was 2 weeks after Christmas so I was quite nervous about getting on those scales. But it went well, and I think because of that I didn’t have any trepidation going back again afterwards. (12025, relational, less deprived, MH)

However, discomfort with attending weight assessments and the experience of feeling stress or anxiety was also quite common. Participants often described feeling shame, embarrassment, or disappointment when attending weight assessments. Even where they indicated that they were ‘*never made to feel that way at all…*’ (13032, relational, more deprived, MH), these feelings were still present. In both SWAT groups, apprehension and fear about attending the weight assessments were connected with negative perceptions about having their weight taken by the researcher and talking to the researcher about their feelings or their weight.

I would say I was probably, like many others, quite nervous for the attendancies [sic]. It’s a topic [weight loss] you don’t really like bringing up…it feels weird to sit and put thoughts and stuff like that out loud and have progress. It was a strange nervousness that went with it…. (11074, task-oriented, more deprived, no MH/MLTC)

Participants in both SWAT groups generally attributed value to attending their follow-up weight assessments. However, they differed in how they described their experiences of attending the follow-up assessments. The task-oriented group generally described their 3- and 6-month weight assessments as functional and brief. For these participants, the purpose of weight assessments was data collection rather than to develop a bond with researchers.

…didn’t expect it to be you know, war and peace, it was only literally a weigh-in and just - how you getting on? and any changes since the last appointment…? if anything I just seen it as an extra scale you know. (13139, task-oriented, least deprived, MH)

Relational group participants were more likely to describe the weight assessments as providing them with a focal point within the trial and they were more than an outcome assessment or trial process. Weight assessments represented a ‘*a good sense check, it was good reset and refresh as well*’ (11009, relational, more deprived, no MH/MLTC). They were viewed as a place for validation and recognition of weight loss efforts which gave a boost or push to the participant’s motivation regarding the BWMI. Receiving reinforcement at weight assessments was not entirely unique to the relational group; however, the centrality of the interaction with the researcher and linking the weight assessment with the intervention was mostly discussed within the relational group.

And the reason for that [focus] is because somebody is gonna make me stand on a set of scales and look about me and say ‘Oh’. So that’s somebody else is going to say well done (P)! You’re fantastic, brilliant…. (11200, relational, less deprived, MH)

##### Sub-theme: the participant-researcher relationship

Participants, in both of the SWAT groups, shared the perception that meeting with the researchers enhanced a sense of accountability in terms of the intervention and trial. Researchers were perceived to be skilled providers, with knowledge, expertise, and the ability to provide support and encouragement. Participants often spoke about the researchers’ inter-personal and relational skills describing them as good listeners, friendly, and approachable. Their compassionate, non-judgemental approach to weight assessments offered reassurance and established trust creating a safe environment which enabled openness. Many participants from both SWAT groups contributed this to the development of a bond (i.e. liking, caring, accepting, trusting).

…I wasn’t being penalised or I wasn’t being told ‘you should’ or, you know, ‘you’re not in the right place’ or ‘you should have been here’ [in relation to weight loss]…It was all…what’s the word I’m trying to think of it, you know, compassion. You know, there was no, there was no judgement. (13166, task-oriented, more deprived, MH and MLTC)

Although the task-oriented group described their interactions as brief, the value of being able to ‘*touch base*’ (11,002, task-oriented, more deprived, no MH/MLTC) with the researchers was considered useful.

They [weight assessments] were very, very simple and straightforward. The girls were always great to be fair and very helpful – get your kit off [heavy outerwear and shoes], get on the scales and just great, and have useful chats…. (11226, task-oriented, less deprived, MH and MLTC)

This contact was an important and helpful part of their overall experience and progress through the trial ‘…*like chalking off a step on the way, you know, on the journey…*’ (12,001, task-oriented, more deprived, MH). A small number of participants in the task-oriented group talked about not having an opportunity to build rapport. However, even the simple act of arranging an appointment with the researcher offered purpose and meaning.

… I think that the fact that I was going in and I was even, even wee things like being set an appointment time meant to me there are other people going through this process. I’m not on my own here. (13035, task-oriented, more deprived, MH)

Participants in the relational group described the openness of the interactions, providing an opportunity to talk through challenges or problems and a sense that participants and researchers were working together.

Nah I mean she [the researcher] was brilliant. I mean if I wanted to sit there longer, she would have sat there and just listened to me. There was no time pressure. So it was absolutely no problem, she was a delight to work with, she really was. (11133, relational, less deprived, MH and MLTC)

These participants also described the ongoing nature of the relationships with the researchers which gave the sense that the researchers knew them and were able to offer emotional support, direction, and help them reframe their difficulties.

The guys in [trial centre] were both fantastic. They were, I suppose as warm as you can be in those circumstances…They were, I suppose non-judgemental. I felt easy to talk about any of the issues that they were asking about… [the researcher] knew who I was and then knew where I was and she was able to point out certain things at the different visits. So that was nice and reassuring. (13007, relational, less deprived, no MH/MLTC)

There were some participants from both SWAT groups who viewed their weight assessments as less meaningful. In the task-oriented group, some participants described how they ‘*didn’t gel*’ (13,185, task-oriented, less deprived, MH/MLTC) with the researchers or the weight assessments were business-like, clinical, or impersonal. For some, this suited their needs.

…it was all business. It was not like we had like any kind of personal chitchat, really, just come and go! Take your weight and go… I was quite happy just to be in and out. (11208, task-oriented, more deprived, no MH/MLTC)

For others in the task-oriented group, the neutral approach described with limited feedback or interaction was challenging. Some participants suggested they disengaged because they did not get what they expected and wanted more from the weight assessments. The trial therefore became a less integral part of their lives. While they understood that the research setting imposed certain limitations, they viewed the ability to develop a deeper bond with researchers as more motivating in relation to connecting with the trial.

…maybe because this was a research thing, you know, everybody’s kind of being very careful…I think it needs to be a bit more personal…. (12155, task-oriented, less deprived, no MH/MLTC)

When participants in the relational SWAT group described how weight assessments had not met their expectations, they talked about not receiving enough encouragement, direction, and support from the researchers in relation to weight management.

…I think they [researchers] were very matter of fact, I think there wasn’t, you know there was a bit of encouragement but there was no, there wasn’t any in-depth analysis of what I was actually doing…. (12147, relational, less deprived, MLTC)

Participants in both SWAT groups perceived that relationships developed with researchers could be a barrier to attendance due to the shame, embarrassment, or disappointment in having a weight taken by the researcher. They thought attendance would be particularly difficult if participants perceived they were not doing well in terms of weight loss and if it was the same researcher. Despite the majority of these participants attending both 3- and 6-month follow-up weight assessments with differing weight loss trajectories, this perception persisted.

…it was a bit embarrassing I had to go to the same people…So I suppose it depends what way you are going on the trial. If you’re flying on it, you definitely want to see the same person again. Do know what I mean? But if you’re not going too well at it, you probably wouldn’t…. (13032, relational, more deprived, MH)

Participants from both SWAT groups described a strong sense of responsibility to the trial and this was motivated attendance. Once committed, many participants were compelled to follow through and play their part. This commitment was strengthened by validation of their efforts by researchers and their relationships with researchers. Participants indicated they did not want to disappoint the researchers by non-attendance.

…I wouldn’t be that disrespectful, and it was very good that way that they were organised and somebody took time out to actually do it [weight assessment] so, and to be honest with you I felt that I was letting her down as well, you know, you turn up. (13185, task-oriented, less deprived, MH and MLTC)

In the task-oriented group, they spoke about commitment in terms of a transactional relationship. There was a perception that if they were being helped it was their duty or role in the relationship to make the effort and give something back, and attendance at follow-up assessments was part of that role.

I always maintain if somebody’s trying to help you or offer you help or advice, you should make the effort or don’t take part, you know. It’s not always one way it’s, you know, it’s to give and take both roads, so you have to do your part as well. (13166, task-oriented, more deprived, MLTC)

Whereas for the relational group, this was less about the relationship with the researcher and more about weight assessments being a necessary part of the trial process. They did not want their time on the trial to be a waste; therefore, they committed to all parts of the trial including engaging with the intervention and attending weight assessments.

…To me if you say you’re going to do a programme, you do the programme whether it works or not…I didn’t commit to losing weight, I committed to try the programme. (12033, relational, more deprived, no MH/MLTC)

#### Theme 2: mode of weight assessment delivery

##### Sub-theme: the human touch

‘The human touch’ encapsulated participants’ views and experiences of having in-person contacts with researchers during the weight assessments. Participants from both the SWAT groups had a strong preference for in-person human contact throughout the trial. In-person contacts were perceived to be fundamental to the trial experience. Participants had an understanding there were greater gains from receiving weight assessments in-person. They believed they enhanced the sense of encouragement, honesty, and acceptance. For many, it made the process more real, improving their connection with the trial.

….it felt more official and more part of a process and part of the project having it done in-person. (11087, relational, less deprived, no MH/MLTC)

Participants from both SWAT groups believed that personalisation, consistency, support, and the sense of being cared for and being part of the trial would be lost without human contact. Positive perceptions of in-person weight assessments were discussed in the context of the widespread emergence of artificial intelligence (AI), digitalisation of healthcare in a post-COVID world which now relied on technology.

…people do need people and people do like people, they like to be around people and like to be measured by people. And yes that’s downsides like everything in life but there’s also massive, massive upsides to a personal thing…there are certain issues that can be solved by technology…. But I think there will always be people that want to talk to somebody about it…. (13173, task-oriented, more deprived, met no targets, MLTC)

The preference for human, in-person connection was partly seen as generational as well as practical. Participants thought that some older people may not be as comfortable with technology and there were some concerns that privacy for remote appointments might be difficult to achieve in busy or shared households.

…if there’s any confidential thing or things that you might feel embarrassed, some people live in the shared accommodations…And I don’t know some people may kind of actually hide the real weight from the partner or from the kids or from other people…So I wouldn’t skip like a face to face meeting at all…. (12182, relational, less deprived, no MH or MLTC)

The benefits of using technology to complete weight assessments were discussed. Participants acknowledged remote methods could be more cost-effective, potentially easier, taking up less time by limiting the need for travel. They also believed remote assessments would impose less pressure, potentially reducing the embarrassment or self-consciousness that was felt during in-person weight assessments.

…If you did it digitally, again, some men might feel more comfortable…I wouldn’t say it’s more comforting, it’s a lot less pressure…. (11243, task-oriented, more deprived, MH)

##### Sub-theme: frequency, regularity, and consistency of interactions

Participants in both SWAT groups had similar views regarding the frequency and regularity of weight assessment interactions during the trial. They differed in their views regarding consistency in relation to having the same researcher throughout their time on the trial.

In both SWAT groups, participants talked about a desire for more frequent contact with researchers. Very few participants supported longer intervals between weight assessments as they believed acknowledgement of effort and rapport would be lost. Participants reported they sometimes felt alone, due to limited contact with the trial. As a consequence, they drifted, forgot about the trial and about attending weight assessments.

… lack of that kind of like booster every like six weeks, 8 weeks…I think I kind of lost after 8 weeks and I missed my first appointment. (12182, relational, less deprived, no MH or MLTC)

Some participants considered the frequency of contacts through the weight assessments as acceptable (i.e. at 3, 6, and 12 months) or would have preferred longer intervals. These participants talked about feeling empowered by the frequency of interactions with the researchers and having space to progress on their own.

… I like being left alone and doing things myself, and motivating myself etc. So for me the 6 months was fine, I didn’t feel like I needed to do it again. I think maybe if I had to go to more of them, I probably would have not wanted to go so often…. (11132, task-oriented, more deprived, No MH/MLTC)

The importance of continuity with researchers was highlighted by both SWAT groups. Having the same researcher at each weight assessment meant that participants knew what to expect, as it gave them a sense of familiarity which helped bonds to develop and personal barriers to be removed.

…walls have to erode and they can only really erode with consistency. (13109, task-oriented, less deprived, no MH/MLTC)

Participants in the task-oriented group particularly emphasised the value of continuity giving a sense of equal investment in the process and working together with the researcher which supported continuing engagement.

…It [having the same researcher] shows that they are involved in it as well. Because you know, if it’s somebody there different every time, you feel like you’re just going through the motions, whereas if it’s the same people, you know, you get the feeling they’re working on it, that they’re dedicated to this all the way through this process. They are with me on this journey. (13035, task-oriented, more deprived, MH)

Participants in the relational group believed having the same researcher was a ‘*bonus*’ but researcher characteristics and having a consistent, standardised approach were more important.

I don’t think it mattered in the respect in that it had to be that person, but I think it mattered in the sense that I knew that the people I was going to see were really nice and easy to talk to and non-judgemental…. (12025, relational, less deprived, MH)

#### Theme 3: contextual and practical factors influencing attendance at weight assessments

There were no clear differences in the main determinants of retention discussed by the two SWAT groups. Both SWAT groups shared similar views about the enablers and barriers related to the impact of personal circumstances, the research setting, the COVID pandemic as well as practical and trial design considerations. Some additional insights from the relational group are highlighted.

##### Sub-theme: enablers of attendance

Some participants who lived or worked locally to where weight assessments were held or had retired from work described greater availability and flexibility to attend weight assessments.

…But most of them I went to them in my lunch break because the GP [location of weight assessment] was near my house, it was like a 5-minute walk…. (11132, task-oriented, more deprived, no MH or MLTC)

Clear reminders of the benefit of attending weight assessments and commitment to the trial were believed to enhance continued attendance.

…Being a bit more harsh with them…I don’t know the ins and outs but if someone commits to something, just remind them that they committed to it… Don’t waste my time…You know, we’re men. We’re not, like, fragile. (11208, task-oriented, more deprived, no MH/MLTC)

Participants in both SWAT groups described the importance of the researcher role as an enabler of attendance. They talked about how researchers offered flexible appointment times, organised convenient venues which were no or low cost to attend, gave lots of notice and reminders for upcoming weight assessments, and were considerate of participant needs (e.g. providing taxis or offering home visits if required).

I couldn’t get to my last appointment because I had no car and [researcher] came out to my house here, she had to travel down from the city and when she went she sat with me and we talked everything through there, you know. She weighed me in the house. (13171, task-oriented, less deprived, MLTC)

Participants believed that having researchers deliver the weight assessments within the trial enhanced trust and made them feel like they were part of something important. A small number of discussions indicated that researchers actively addressed withdrawals and supported participants to attend by offering information and reassurance which prevented loss to follow-up or drop-out.

…she [the researcher] reined me back in a couple of times, you know there were a few times where I was fairly, you know, just as I say, jacking it up and she says no, come along sure and we’ll chat and we’ll check your weight and do whatever…. (13120, relational, more deprived, MLTC)

Having a clear boundary between research and healthcare contexts was viewed as helpful. Participants reported that knowing weight assessments delivered in the trial were different from those delivered by healthcare professionals or within weight loss groups enabled attendance. This was connected to beliefs that the trial offered a more professional, respectful, and credible setting.

I think in my case it being researchers, it being academic, it being part of a study actually was quite beneficial. I think it needed to have a certain amount of medical, clinical reputation behind it. Someone you know is going to be impartial, trustworthy, hasn’t got an ulterior motive…. (12014, relational, more deprived, no MH/MLTC)

This trial was conducted in the context of the COVID pandemic. Despite restrictions still being in place, there was some indication that participants, from both groups, still felt safe coming to their appointments. A few participants viewed the weight assessments as a welcome opportunity to travel and socialise during a period where interactions with others were limited.

…It was good to get back into [local city] after a while because some of it was still during the…there were still some lockdown restrictions at the start of process so…. (13035, task-oriented, more deprived, MH)

For participants in the relational group, experiencing positive interactions with researchers during the weight assessments alleviated fears and helped participants to make the separation between usual healthcare and research. Participants talked about how attending one weight assessment encouraged further attendance.

The first one, that was…I was apprehensive and then once I sat down with [the researcher] and she spoke through things and I got my weight done and I felt a lot more comfortable with things. (11133, relational, less deprived, MH and MLTC)

Participants in the relational group had several suggestions about how to improve trial design. They believed that attendance could be further enhanced by incentivising attendance and with the opportunity to speak to other participants who had experience of attending the weight assessments. They thought speaking to other participants could reinforce the idea that this was a non-judgemental environment, offer them reassurance, and therefore encourage them to attend follow-up assessments.

…speak to somebody else who’s doing the programme…You know, just to sort of say – nah, there’s nothing to it, they don’t judge, you have a natter, stand on the scales…. (12157, relational, less deprived, MLTC)

##### Sub-theme: barriers to attendance

Life circumstances such as family responsibilities and holidays as well as physical health problems were major barriers to attendance. A small number of participants interviewed, from both SWAT groups, were unable to attend each of their follow-up weight assessments due to ongoing health problems, such as injuries or medical diagnoses, as attending weight assessments became less of a priority.

Well I had health issues about half way through I was diagnosed with the cancer so I kind of dropped the ball so to speak, so it was maybe four or five months into it, I was doing quite well but that just hit me for six and I kinda just lost focus and maybe a certain amount of interest in it to be honest. (13080, task-oriented, less deprived, no MH or MLTC)

A strong connection was drawn between retention and perceptions about weight loss outcomes. There was a belief that participants would drop-out if they saw themselves as unsuccessful in relation to weight loss. These perceptions regarding weight loss were linked to feelings of shame or embarrassment which participants also viewed as a barrier to attendance.

When the head goes down that’s possibly why a lot of men, some men, didn’t come back because they seen their weight going [up]. (13171, task-oriented, less deprived, MLTC)

In this research context, weight loss and retention were also interconnected with participants’ misunderstanding the purpose of trial processes including the weight assessments. Some participants believed that they would negatively impact the trial if they attended their assessment without having lost weight or if they were less successful within the trial, they were preventing someone else from taking part who could potentially benefit. This made them question if they should attend at all.

…I knew I hadn’t lost weight, or not enough weight, you know…I actually said to [the researcher]…I don’t want for someone to stay on the waiting list even that really maybe wants to avail of the opportunity…. (13120, relational, more deprived, MLTC)

Gendered views were described as potential barriers to attendance at weight assessments. Some participants believed that men are more reluctant to attend healthcare appointments than women and based on this suggested that men would actively avoid weight assessments in particular if the perception was that these were comparable to attending healthcare appointments or weight loss groups.

if you can motivate men, especially middle aged and seniors to turn up to a medical centre you’re a good’un…blokes are just a completely different mindset, you know? (13139, task-oriented, less deprived, MH)

In addition, they suggested that male participants may have difficulties interacting with a female researcher (the majority of researchers were females across centres) and this could also negatively impact attendance.

…When you’re interacting with a female host [researcher] some of them [participants] would be uncomfortable with the shift of power so they would, you know that general switch of power. (13185, task-oriented, less deprived, MH and MLTC)

Some discomfort about attending follow-up assessments was also discussed in the context of the COVID pandemic. This was a period of various levels of lock-down with changing rules and circumstances. These participants described their unease with attending weight assessments in small rooms where everyone had to wear masks. They questioned attendance at research appointments when restrictions were in place whereas people generally could not attend healthcare appointments in-person.

…but you’re masked in a tiny room so no-one’s quite comfortable (laughing) sitting there going – should I even be in this room? (13109, task-oriented, less deprived, no MH or MLTC)

The research setting was also discussed more generally as a barrier. Participants spoke about limited parking, difficulties navigating unfamiliar locations (e.g. university campuses), accessibility of the buildings, travelling long distances, and inconvenient appointment times. Attending weight assessments was particularly difficult for participants who were working. This was a further constraint impacting availability and making weight assessments harder to arrange. In terms of trial design, there was also a suggestion that the way in which people were reimbursed for out-of-pocket expenses by receiving a voucher rather than cash was prohibitive.

## Discussion

This qualitative study offers novel insights into retention, specifically attendance at follow-up weight assessments within a BWMI trial, from the perspective of participants. Descriptions of follow-up weight assessments differed between the SWAT groups; however, they offered value for many participants irrespective of the approach received. This was demonstrated in the broadly similar participant views and experiences related to attendance were described from both SWAT groups across each of the three themes: the value of attending follow-up weight assessments, the mode of weight assessment delivery, and contextual and practical factors influencing attendance at weight assessments.

A number of key determinants of attendance at follow-up assessments were identified based mainly around the participant-researcher relationship, the research context including the researcher role as well as logistical and health-related factors. Participant-trial staff relationships have shown promise in previous studies [26–29]; however, there has been an indication this could be difficult to implement [29]. Our findings particularly reflect evidence from a recent qualitative study of participant perspectives to understand difficulties with retention in trials [29]. Here the authors mapped behaviours specific to attending follow-up clinics to the Theoretical Domains Framework (TDF). They identified several key domains including the environmental context and resources (e.g. work commitments or travel time), social influences (e.g. the demeanour of trial staff), and emotion (e.g. feeling stressed or anxious about attending).

Our qualitative study builds on this evidence demonstrating that participant-researcher relationships were important to participants in both SWAT groups and offered mutual benefit for the participant and for trial retention. On the one hand, participants valued the interaction with researchers because they gained encouragement, validation, and reinforcement. This relationship also reduced anxieties about attending and enhanced commitment to the trial, and participant motivation to continue to attend. However, although this relationship was shown to be an important pull in terms of attendance, there was an understanding that it could also push some participants away from attending.

Participants described conflicting feelings and thoughts about attending related to perceived failings in relation to weight loss outcomes. Feelings of shame or embarrassment regarding attending weight assessments were commonly expressed as well as worries about disappointing the researcher both in terms of not losing weight but also non-attendance. The negative feelings about attending weight assessments were reflective of lived experience of weight stigma conceptualised both in terms of self-devaluation (internalising negative beliefs about themselves) and fear of enacted stigma (the fear that others will have negative attitudes, judge, and discriminate against them) [30]. The participant-researcher relationship could have an important role within trial delivery to create a safe environment for participants, reducing stigmatising experiences for those living with obesity thereby improving retention.

Perceptions regarding weight loss were also inter-related with uncertainties about participation in the trial which in turn impacted attendance. Participants were often concerned about negatively impacting the trial or other participants by continuing to attend without sufficient weight loss. Recent research has demonstrated that participants can have difficulties understanding key concepts within trials [31]. Conflation of weight assessments with the BWMI was a further indication of struggles to understand participation and to distinguish between the trial and the intervention. This suggests that better understanding of trial conduct could improve retention in particular where trial processes, such as weight assessments, may also reflect components of an intervention or closely align with usual care.

Based on evidence from a previous feasibility study [32], the host trial was designed to have minimal in-person contact. The intention behind having a small number of follow-up assessments was to encourage participation from men, who were the target population, for whom engagement in weight loss trials has been more challenging [33]. However, participants generally discussed the desire for more frequent follow-up weight assessments and viewed this as important to stay connected to the trial. In addition, continuity with researchers was also considered valuable and participants had a preference for in-person interactions over remote weight assessments. Some recognised the practical benefits of receiving weight assessments remotely. However, it was clear that participants believed that in-person enhanced motivation to continue to attend. In contrast to the feasibility study, the SWAT was carried out during the COVID-19 pandemic at a time when face-to-face contact with healthcare professionals was very limited [34]. The opportunity for human contact may have been given greater value because healthcare interactions were very limited or unavailable. Implementation of in-person interactions and consistent ongoing relationships has implications for trial conduct. This reinforces the need for a considered approach to the adaptation of trial processes with sensitivity to the needs of participants and contextual factors [35].

Participants perceived the research setting as credible and researchers trustworthy sometimes in contrast to usual healthcare settings. Researchers were also viewed as having a central role in enabling attendance within the research setting. They were in a position to consider participants’ needs and make adjustments to delivery of weight assessments to suit those needs. There did not appear to be a connection between deprivation or living with mental health problems or multiple long-term conditions and attendance. Moreover, the main reason for non-attendance was experiences of physical health problems during the trial such as new medical diagnoses or injuries coinciding with follow-up weight assessments. Medical concerns regarding physical health have been shown to motivate engagement to join weight loss programmes and trials [1]. However, struggles with physical health can become a barrier to retention. Trial design should consider health-related factors in particular for trials such as Game of Stones with specific follow-up requirements and high levels of multi-morbidity [22].

### Strengths and limitations

By embedding this study within a host trial, we aimed to maximise knowledge of trial conduct, in this case retention, while reducing burden and research waste [36]. Many aspects of the SWAT therefore relied upon or were determined by the host trial which took precedence. This somewhat constrained aspects of the embedded SWAT. There was for example an imbalance between the SWAT groups which may have resulted in some voices, in particular those from the task-oriented group and non-attenders, being underrepresented. However, 54 men took part and consideration of information power throughout helped ensure that the sample was closely monitored and a diverse group of participants from SWAT groups were selected.

The focus of this qualitative study was upon attendance at follow-up assessments with data predominantly from attenders. Although other important aspects of retention such as withdrawals or drop-out were discussed, these had less attention. The narratives captured provide important information about retention in relation to men taking part in a text-based BWMI trial. The results therefore presented insight into retention within a stigmatised population and could be reflective of other studies in this area [29, 37] offering potential transferability into other trial contexts.

This exploration of attendance behaviour supports understanding of what motivated participants to take action and continue to engage for the duration of this BWMI trial [8] and advance understanding of what works and for whom. Identifying the determinants of attendance can help to develop evidence-based strategies by better understanding the active ingredients to address barriers and enhance the enablers of this aspect of retention [38]. Through this behavioural lens, we aim to contribute to the growing evidence-base progressing improvements in trial conduct [28, 39, 40].

### Research recommendations

From the participant narratives, the two SWAT groups had distinguishable characteristics. However, participants in both SWAT groups also described developing relationships with researchers and experiencing interactions within weight assessments which went beyond a trial outcome assessment. This suggests potential difficulties with protocol adherence. Implementation fidelity (understanding if an intervention was delivered as was intended) is a factor which can influence the relationship between an intervention and its intended outcome [41]. Future research of retention strategies should incorporate fidelity as a critical aspect of understanding why a strategy works or not and contributes to the desired outcome. Obesity trials should clearly consider how retention strategies can sensitively mitigate the complexity of weight stigma as well as experiences of poor health. Participant-researcher relationships can be influential in clinical trial conduct. We believe this should form part of any critical appraisal similar to how this is considered in assessment of qualitative studies such as through the Critical Appraisal Skills Programme (CASP) qualitative checklist [42].

## Conclusion

Participant-researcher relationships within trial processes have an important role in attendance and offer a potential retention strategy, which to date has been largely overlooked in trials and SWATs. The approach of researchers in outcome assessments mattered by supporting motivation through mechanisms of change such as engendering a sense of responsibility or accountability. Removal of relatedness through digital-only interventions or during participant follow-up is likely to reduce retention and have implications for wider intervention implementation in real-world settings.

## Supplementary Information

Additional file 1: COREQ.

Additional file 2: Guidance for researchers.

Additional file 3: Qualitative sub-study patient information leaflet (PIL).

Additional file 4: 12M interview topic guide.

Additional file 5: Stages of framework method in practice.

Additional file 6: Qualitative interviews code book.

## Data Availability

The qualitative data collected for the study, including anonymised individual patient data and a data dictionary defining each field in the data set, will be made available to others by reasonable request. The participant data will be de-identified and will comply with the ethical and regulatory approvals for the study. Requests for the datasets used and/or analysed during the current study will be considered by the study team through the corresponding author.

## Abbreviations

BWMI: Behavioural weight management intervention
COREQ: Consolidated criteria for reporting qualitative research
MH: Mental health
MLTC: Multiple long-term conditions
SWAT: Study within a trial
CASP: Critical Appraisal Skills Programme
