# Supplementary material for: ‘They are with me on this journey’, the importance of participant-researcher relationships on attendance at follow-up in a behavioural weight management trial: a qualitative study of participant perspectives
Source: Trials. 2026 Jun 6;27:521. doi: 10.1186/s13063-026-09818-w (PMC13459619; doi:10.1186/s13063-026-09818-w)
Supplement: Supplementary file 2 [file 13063_2026_9818_MOESM2_ESM.docx]

**Guidance for Fieldworkers**

**Part 2 – Follow-up Assessment Visits**

| **Section** | **Contents** | **Page(s)** |
| --- | --- | --- |
| **1.** | 3 and 6 Months Assessment Visits (Intervention Groups Only) | **1-3** |
| **2.** | SWAT Weight Assessment Protocols | **3-4** |
| **2.1** | Task-Oriented Group | **3** |
| **2.2** | Relational Group | **3-4** |
| **3.** | Discussing Follow-up Weight Targets and Incentives | **4-5** |
| **3.1** | Intervention Groups | **4** |
| **3.2** | Waiting List Control Groups | **4-5** |
| **4.** | Completing follow-up questionnaires (12M &24M) | **5-6** |
| **5.** | 12 and 24 Months Assessment Visit | **7** |

Part 2 of the guidance for fieldworkers is designed to help you carry out your role in relation to follow-up assessment visits. Please see Appendix 1 Guidance for Fieldworkers Part 1 for additional information on carrying out assessment visits.

This document is supplementary to and should be read alongside SOP1, SOP3 and SOP4. There are certain tasks, discussions and protocols that must be carried out or followed in this role. These are highlighted in bold throughout this document. We have offered suggestions on how to approach tasks and discussions however the delivery is up to each individual fieldworker at each site.

# 1. 3 and 6 Month Weight Assessment Visits (Intervention Groups only)

| 3 and 6 Months Assessment Visit Checklist: | |
| --- | --- |
| Equipment | Scales |
|  | BMI Calculator |
|  | Baseboard |
|  | Shoe-horn |
|  | Mobile phone (charged) and charger (leave switched on during the visit) |
|  | Charged tablet computer and charger |
|  | Audio-recorder (Charged) |
|  | Pens |
| Paperwork | CRF (must be completed on paper) |
|  | Researcher 3 Months Evaluation |
|  | Researcher 6 Months Evaluation |
|  | Appointment cards |
|  | Fieldnotes (Excel/ Notepad) |

Please note there is no participant questionnaire to be completed at 3 and 6 months (only the CRF paperwork is required).

**Preparation:**

When arranging the 3 or 6 months assessment visits remind men that their appointment will be recorded for research purposes (as agreed during consent process) and this will be a much shorter appointment.

**IMPORTANT:** Before visits **check the allocated SWAT weight assessment group** for each man reported on HIC tracker/ CHaRT database, in order to follow the correct protocol (see section 4). Fieldworkers will also have received an email regarding the allocated SWAT group at recruitment. **Do not disclose the SWAT assessment group to men.** If men are unblinded this should be noted in the 3 and 6 months CRF**.** See section 2 for further information on how to complete the 3 and 6 months weight assessment protocols.

**During Weight Assessment Visits:**

**IMPORTANT: Lead fieldworkers (not fieldwork assistants) will carry out 3 and 6 months assessment appointments. This should be the fieldworker who led the baseline assessment visit. The fieldworker assisting should carry out the weight measurement on research scales.**

- **Before beginning the weight assessments at 3 and 6 months participants should be reminded that the assessment will be audio-recorded** (this is for research purposes and was agreed during consent). There may be occasions when men may refuse to be recorded and this does not affect undertaking the 3 and 6 month appointments and fieldworker evaluations (an audio-recording would not be submitted).
- **Please state the Participant ID, Fieldworker ID(s), Initials and date clearly at the start of the recording (e.g. this is participant ID Xxx, fieldworker ID Xxx, Assisting Fieldworker ID Xxx and the date is the XX/XX/XXXX).**
- **Remember to record the start and finish time for weight assessments on the CRF.**
- **Remind the man not to disclose his allocated group.**

**After Weight Assessment Visits at 3 and 6 months:**

- **Audio-recordings (with fieldworker ID, participant ID, numbered from 1 onwards and dated e.g. F01_90019_01_29.8.2021) should be submitted as soon as possible after appointments** by uploading to the fieldworker’s individual audio-recording folder (3 or 6 months) in SharePoint site: Game of Stones Fieldworker Evaluations and Fieldnotes (Document Folder: 3&6M Evaluations: Audio-Recordings). Fieldworkers have access to this via email or Microsoft Teams– any issues contact Claire Torrens.
- **The 3 or 6 months researcher evaluation should be completed after each assessment visit,** as appropriate, these can be completed electronically and uploaded to the fieldworker’s individual evaluation folder in SharePoint (Folder: 3&6M Evaluations: Evaluations). Please make sure researcher initials and Participant ID number are recorded at the top of each page and keep any paper copies in the local site file.
- **Fieldworkers should also record two ratings for each audio-recording**: the quality of the relationship and adhering to the protocol of the submission 0 (worst it could be) -100 (best it could be). This should be completed on the SPSS database on SharePoint (Folder: 3&6M Evaluations: Audio-Recordings: Audio-Recordings_Evaluation). If fieldworkers cannot access SPSS there is an excel database in the same folder as a back-up.

**Label evaluation**

**When uploaded, complete ‘Fieldworker Evaluation_Audio-recordings_9.4.2021.xlsx with details**

**Label recording**

# 2. Weight Assessment Visit Protocols (Intervention Groups Only)

**The following Protocols must be followed at 3 and 6 months.**

**IMPORTANT: Please ensure you are clear which protocol to follow before each assessment visit:**

In both groups thank the men for their time and for participating in the research.

## 2.1 Task-Oriented Group

This contact should only allow for the completion of the necessary weight assessment tasks (highlighted) with time and discussion kept to a minimum.

1. **Take the weight measurement on research scales (the man must verify this by initialing the relevant box) following the weight protocol (See Guidance for Fieldworkers Part 1).**
2. **Complete the CRF including recording any adverse events or unintended consequences, check contact details are correct and ensure participant has initialed the weight taken. Any changes to their contact details should be updated on the HIC tracker.**
3. **Refer the man to their weight targets.** Fieldworkers can update this on their appointment card. Note: For the incentive group only, fieldworkers should explain that a text (also sent by email and on personal pages) will inform them of any money lost (computer calculates) (see Section 3).
4. Remind participants that it is important that they attend all follow-up appointments no matter what happens with their weight (See Guidance for Fieldworkers Part 1 – Assessment Visits)
5. **If the man has any questions about the research processes fieldworkers can answer these (i.e. appointment dates/ times, allocated group).** Fieldworkers can also direct them to the website or Game of Stones Team if they need specific help.

**In this group in task 5 if questions are about how to lose weight, health or wellbeing – explain that you are not in a position to answer those types of questions** (e.g. “I can’t really answer that...what might be helpful is...to go back to the texts/ get in touch with the trial team etc)”) **and direct them to the website, texts or Game of Stones Team for further information and advice rather than answering these.**

## 2.2 Relational Group

This contact should allow for the completion of the weight assessment tasks 1-5 above and also help develop the fieldworker-participant relationship. **The approach in this group is underpinned by the core conditions (i.e. understanding the participant (empathy), being genuine and non-judgemental (warmth/ respect)) and the use of fundamental interpersonal skills (e.g. active listening, minimal encouragers, reflecting, summarising and being interested in the participant).**

- **Complete tasks 1-5**

**In this group in task 5 participant-centered questions (i.e. directed by the man) and discussion are welcome (see below).**

**AND**

6. **If the man has not initiated discussion or asked questions, ask an open ended question about how they have been doing and if there was anything they would like to talk about?** (e.g. How have things been over the past few months? Was there anything you wanted to talk about today?”)
7. **Actively listen to anything they might want to discuss including (but not limited to) concerns about the programme, their weight or other challenges they are facing. Provide space for them to talk, a listening ear, support and information as necessary** (men can be directed back to the texts, website or Game of Stones team as appropriate).

# 3. Discussing Follow-up Weight Targets and Incentives

## 3.1 Intervention Groups:

- Weight targets will be discussed with men at each of their assessment visits (see SOP3 and SOP4 for further information regarding follow-up visits)
- After follow-up appointment visits (3, 6 and12 months) men will be told by a text message (within ~ an hour of their appointment time) and on their personal web page whether they have met their target or not. They will also be reminded of what their next target is and when their next weight assessment will be (Fieldworkers do not calculate this).
- At 12 months follow-up assessment visit men will be invited to set their own target for the following 12 months. This can be reviewed at their 24 months assessment visit if they wish.

***Incentive Group Only:***

- Men in the incentive group will be provided with a text message explanation of what meeting/ not meeting targets means in terms of incentives (see SOP3 and Guidance for Fieldworkers Part 1 - Post-Randomisation Discussions)
- Not met target – money (£Xxx) has not been secured. However, they can still secure the remaining money if they meet their next target (e.g. at 6 and/ or 12 months).
- Met target – money (£Xxx) secured
- Remind men if they **put all of the weight lost back on and weigh more at 12 months than they did at baseline (even if they have met their 3 and 6 months targets), they will lose all of the money.**
- **We will do our best to arrange a time, date, venue for this assessment within 4 weeks (2 weeks before and 2 weeks after the assessment date) to ensure an accurate weight record.**
- A computer works out the weight targets and dates. **Research scales must be used and are the only ones that count for incentives.**
- Fieldworkers verify the weight with men i.e. agree the weight recorded is correct on entry to the database but other than recording this on their appointment card they have nothing further to do with the weight targets or payments,
- Money is transferred securely at the end of the programme, vouchers may be available as an alternative if preferred (the process should be determined at each centre),
- **Remind men not to disclose the amount of the incentive to other men taking part in Game of Stones or on social media**
- At 12 months fieldworkers should discuss the transfer of money relevant to the cash incentives gained. This discussion will depend on the process at each centre – please refer to Appendix Xxx for guidance on incentives payments and procedures (TBC).

3.2 Waiting List Control Group

- At 12 months men in the waiting list control group will be invited to set their own weight target for the following 12 months. Any weight targets set should be recorded and can be reviewed at their 24 months assessment visit if they wish (see section 5 – waiting list control)
- Men in this group may not have thought about a target weight so it may be useful to use the following: “*would you like to set a weight target for when we meet again in 12 months time? I’ll record it for you and we can review it next time if you like. This is just for your own personal progress”.*
- Fieldworkers can discuss targets in line with health recommendations and those set with the intervention groups:
- 5% to be achieved by 3 months,
- 10% to be achieved by 6 months and
- 10% weight loss to be maintained at 12months.

Note: These targets were set because losing between 5% and 10% has been shown to have considerable benefits for health (e.g. lower blood sugars, lower blood pressure and reduced risk of heart disease, fewer knee and hip problems, feeling healthier, fewer hospital admissions)

- Men in the control group do not have to follow the recommended weight loss for health benefits their target weight is their personal choice.

# 4. Completing Follow-up Questionnaires (12 and 24 Month Assessment Visits)

***All Groups:***

- **Questionnaires at 12 and 24 months should be completed before weight is recorded** (e.g. ‘*would you mind completing this questionnaire first and then I/ my colleague will take your weight?’*),
- Use the tablet where possible. If there is no internet or other difficulties arise (e.g. men insist/ prefer fieldworkers complete the questionnaire) use a paper copy. This should then be entered onto the database after the appointment, with the paper copy stored securely.
- Men can separately log-in to complete the questionnaires at 12 and 24 months (see SOP2 and tracker and database guidance).
- Due to the sensitive nature of the questionnaire, the man should be given as much privacy as possible while ensuring the Fieldworker is on hand to help or answer any questions.
- Explain to the man that it is important to the research that they provide an answer to every question if possible – there are no right or wrong answers, they should answer as honestly as possible.
- They will not be identifiable to the independent researchers who analyse the questionnaire.
- Questionnaires should be checked carefully (after the tablet is handed back or on paper) for missing data and all fields are correctly completed before the man leaves.
- Explain to the man that if any problems come to light with completion of the questionnaire i.e. lots of missing data, the Fieldworker may phone them to arrange sending out a paper version or to complete over the phone.

***Intervention groups only:***

- At 12 months visits participants in the intervention groups will also complete the CARE measure as part of their 12 months questionnaire**. Fieldworkers should inform participants that this should be completed based on their thoughts/ understanding of the approach they received the 3 and 6 month appointment visits rather than the individual researcher.** This will account for circumstances where consistency of fieldworker was unable to be maintained.

# 5. 12 and 24 Months Assessment Visit

| 12 Month Assessment Visit Checklist : | |
| --- | --- |
| Equipment | Scales |
|  | BMI Calculator |
|  | Baseboard |
|  | Shoe-horn |
|  | Mobile phone (charged) and charger (leave switched on during the visit) |
|  | Charged tablet computer and charger |
|  | Pens |
| Paperwork | CRF (must be completed on paper) |
|  | 12 or 24 Month Questionnaires |
|  | Appointment cards |
|  | Qualitative Sub-Study PIL (for selected men) |
|  | Fieldnotes (Excel/ Notepad) |

**Preparation 12 and 24 month assessment visits:**

- Before visits check with the Qualitative RF (CT) which men should be approached for interviews.
- **Work as a team to try to plan to keep one of the main fieldworkers blind to group allocation**. Fieldwork assistants can assist carrying out the weight outcome assessment to ensure outcome assessment blinding (see SOP2 and SOP3). If unblinding occurs this should be noted on the 12 or 24 month CRF.

**During 12 and 24 month assessment visits:**

***All groups:***

- Remind the man not to disclose his allocated group (to keep the fieldwork assistant blind to the allocation),
- Complete the relevant questionnaire (see section 4),
- Complete the relevant CRF, including the weight assessment, once weight completed and initialed by participant – blinding to group allocation is no longer required,
- Confirm arrangements for £20 gift voucher for attending these appointments

***Intervention Groups only:***

- Discuss the qualitative sub-study with men selected (purposive sampling), answer any questions and give men the PIL to take away. Remind them of providing consent at their baseline appointment, this is voluntary, we are keen to understand men’s views about the programme, what worked/ didn’t work for them which will help us to develop Game of Stones further and make sure it fits men’s needs.,
- Confirm consent to be interviewed (if identified by Qualitative RF) and add to HIC 12 or 24 month appointment notes.

**During 12 Months assessment visit:**

***All Groups:***

- Men can set a 24 month target weight, if they wish (see section 3),
- Participants should be reminded of the importance of attending their final 24 month appointment in 12 months time no matter how their weight loss is going,
- Remind men they will receive a £20 gift voucher for attending their next appointment.

***Waiting List Control Group:***

Note: The 12 months assessment visits will be the first appointment since baseline for the men in the waiting list control group.

**Follow the steps outlined above for all groups with the addition of the following:**

- If men in the waiting list control group would like to set their own target weight over the next 12 months see section 4 – Discussing Weight targets and incentives.
- Men will be informed they will receive text messages over the next 3 months. Fieldworkers should follow the process for receiving automated texts (see Part 1 Guidance for Fieldworkers – post-randomisation discussions).
- Ensure the mobile phone number is correct.
- Ensure that participant status on the HIC tracker is updated to Control group – Texts commenced.

**During the 24 month visit:**

*All groups:*

- If men had set a weight target at their 12 months assessment visit, discuss this with them relating it to further weight loss, weight gain or weight maintenance e.g. *“I can see from your notes that you had suggested a target weight of Xxx kg/ stones. From taking your weight today you’ve lost/ gained/ maintained your weight over the last 12 months. Is that what you expected? What do you think are your next steps?”*
- Confirm arrangements for receiving a £20 gift voucher for attending this appointment,
- Discuss consent for linking to health outcomes e.g. hospital admissions and future contact about the findings (TBC).
- Fieldworkers should ask for a postal address and add to HIC tracker if this has not already been recorded for future contact.
