# Supplementary material for: ‘They are with me on this journey’, the importance of participant-researcher relationships on attendance at follow-up in a behavioural weight management trial: a qualitative study of participant perspectives
Source: Trials. 2026 Jun 6;27:521. doi: 10.1186/s13063-026-09818-w (PMC13459619; doi:10.1186/s13063-026-09818-w)
Supplement: Supplementary file 3 [file 13063_2026_9818_MOESM3_ESM.docx]

**Game of Stones:**

**Helping Men to Lose Weight**

**Interviews about your experiences**

Thank you for taking part in Game of Stones. We would like to invite you to take part in an interview about your experiences. This interview is optional you can still remain in the Game of Stones research if you prefer not to take part. Before you decide, we would like you to understand what it would involve for you and answer any questions you have. Talk to others about the research if you wish and please ask us if there is anything that is not clear.

**The purpose of the interviews**

The purpose of the interviews are to find out how men found taking part in Game of Stones. We want to know what it was like to receive text messages and whether they helped men to make changes. We also want to know whether there was anything that helped or hindered taking part, and your views about what we could do differently. We would like to speak to men from each of the different areas taking part to get different viewpoints.

**Why have I been invited?**

You have been invited to take part because you have been receiving text messages as part of Game of Stones. As you are no longer receiving text messages we would like to ask you about your experience of taking part. This is a chance to tell us your views on the text messages and Game of Stones as a whole.

**Do I have to take part?**

No, it is your decision about whether or not you wish to take part in an interview. If you agree to take part and then change your mind, you can withdraw at any time without giving a reason. Your decision will not affect you or your participation in the Game of Stones Trial.

**What will happen if I take part?**

A researcher will contact you to arrange a convenient time to set up the interview, which will take place over the telephone or using another remote device (e.g. Microsoft Teams) whichever you prefer. They will answer any questions you might have about taking part. The interview will last no more than 45 minutes and will be audio-recorded, so that the researcher does not have to take notes. The researcher will ask you to say at the start of the interview that you are happy to take part in the discussion. If you have any questions before you make a decision, about whether or not to take part in an interview, you can contact the contact the Game of Stones research team (details overleaf).

**What are the benefits of taking part and are there any disadvantages?**

There is no extra benefit to you if you take part in an interview. However by taking part you will get an opportunity to tell us your views and you will help us to better understand how Game of Stones works or does not work for men and what changes we can make to improve it.

**Will my participation be confidential?**

Yes. Any information that you give us will be kept confidential and will be stored securely on password protected computers and in locked filing cabinets at the participating Universities (Aberdeen, Belfast, Bristol, Glasgow Caledonian, Stirling and Dundee). If a researcher believes that you have told them something that places you or others at serious risk of harm, then the researcher is obliged to pass this information on to someone who could help, for example your GP.

**Who is organising and funding the research?**

Game of Stones is happening in specific areas e.g. Glasgow, Bristol, and Belfast. Members of the public have helped us to design Game of Stones and will continue to advise us. The research team is led by Professor Pat Hoddinott who is based at the University of Stirling. Research experts from the Universities in Aberdeen, Belfast, Bristol, Dundee, and Glasgow, are also involved. Representatives from the Men’s Health Forum and Men’s Sheds are supporting our research. Game of Stones is funded by the National Institute of Health Research.

**Who has reviewed Game of Stones?**

Game of Stones has been reviewed by the North of Scotland (2) Research Ethics Committee.

**How will we use information about you?**

For these interviews, we will need to use information provided by you including your name, information you provide on research questionnaires and contact details. People will use this information to do the research or to check your records to make sure that the research is being done properly. People who do not need to know who you are will not be able to see your name or contact details. Your data will have a code number instead. We will keep all information about you safe and secure. Once we have finished the research, we will keep some of the data so we can check the results. We will write our reports in a way that no-one can work out that you took part in the research.

**What are your choices about how your information is used?**

You can stop being part of the research at any time, without giving a reason, but we will keep information about you that we already have. If you choose to stop taking part in Game of Stones, we would like to continue collecting information about your health from [central NHS records/ your hospital/ your GP]. If you do not want this to happen, tell us and we will stop. We need to manage your records in specific ways for the research to be reliable. This means that we won’t be able to let you see or change the information we hold about you.

**Where can you find out more about how your information is used?**

You can find out more about how we use your information by asking a member of the research team or contacting Stirling University’s Data Protection Officer, Joanna Morrow: [data.protection@stir.ac.uk](mailto:data.protection@stir.ac.uk). More information on how data is used in research can be obtained from [www.hra.nhs.uk/patientdataandresearch](http://www.hra.nhs.uk/patientdataandresearch). The researcher can give you a paper copy of this information if you prefer.

**How do I contact you?**

**For more information you can phone or text a member of the research team on 01786 467491, email at gameofstones@stir.ac.uk or visit the website www.gameofstonesresearch.com. The administration team can also be contacted at 01786 466341.**

**If you would like to speak to a University member of staff independent to the research please contact Jayne Donaldson by phone 07904635881, email** [fhss.dean.pa@stir.ac.uk](mailto:fhss.dean.pa@stir.ac.uk) **or by post: 4B157, University of Stirling, Stirling FK9 44A.**

**If you need additional help – we suggest the following:**

The NHS choices website: <http://www.nhs.uk/LiveWell/Loseweight/Pages/Loseweighthome.aspx>

The Men’s Health Forum: <https://www.menshealthforum.org.uk>

Or if you need a listening ear for your problems, phone The Samaritans on 116 123. They are available 24 hours a day, 365 days a year.
