# Supplementary material for: ‘They are with me on this journey’, the importance of participant-researcher relationships on attendance at follow-up in a behavioural weight management trial: a qualitative study of participant perspectives
Source: Trials. 2026 Jun 6;27:521. doi: 10.1186/s13063-026-09818-w (PMC13459619; doi:10.1186/s13063-026-09818-w)
Supplement: Supplementary file 4 [file 13063_2026_9818_MOESM4_ESM.docx]

**12 Month Interview Topic Guide (SMS only and SMS+I Groups)**

**12M Questions and Prompts**

‘I would like to have a chat with you about your experience of Game of Stones.’

**General**

- **How have you found taking part in Game of Stones?**

(Probe further around their experience (usefulness) of the texts, the follow ups, the trial and incentives (incentive group)

**Prompts:**

- have there been any benefits for you from taking part in Game of Stones?
- Any benefits that you didn’t expect?
- Have there been any downsides to it?
- how did you feel about the weight-loss targets?
- were the targets realistic for you? (Or probe around other words they use to describe targets)
- if a friend was thinking of joining Game of Stones, what would you say to them?
- **Have you any suggestions about how Game of Stones, or parts of it, could be improved?**

***If not already covered:**

- **What about…the duration (texts stopping after 12M) …the texts …the website …incentives …getting to know other men?**
- **Has anything made it difficult for you to take part in Game of Stones?**

*NB Examples of key words to probe further: stress, mental health, physical health, failure, shame, medicines, body image, self-esteem, relationships (e.g. you talked about Xxx can you tell me a bit more about that/ the impact that’s had generally/ game of stones?)

**Prompts:** pandemic (What kind of impact has Covid or the pandemic had on the Game of Stones for you?); cost of living crisis (What about the rises in costs that have happened recently – how have they impacted Game of Stones for you?)

**3 and 6 Month Follow-up Appointments**

- **Thinking back to your 3 and 6 months appointments – can you tell me about what they were like?**

**Prompts:**

- How were you on the run-up to these appointments?
- Did your behaviour or motivation change, before or after these appointments?
- How was the length of the appointment?
- **What did you think about the appointments being face-to-face?**

**Prompts:**

- Some men have had contact with the same researcher throughout Game of Stones and for others it’s been different researchers – what was your experience?
- Probe depending on experience (how did you feel about the fact it was the same person throughout?/ how did you feel about the fact it was different people?)
- Would you change anything about these e.g. when they happened/ timeframe or who carried them out?
- **Some men found it difficult to attend appointments, did anything get in the way of you coming to both of your 3M and 6M appointments?**

**Prompts:**

- If yes, what would have helped you to attend?
- What might encourage other men to attend appointments?

**In future, it might be difficult to have appointments for everyone, or at all times. Do you have any suggestions about what we might do instead?**

**Prompts:**

How do you think that would work?

What would the benefits/downsides be?

What would you think about replacing these face-to-face appointments with digital scales?

**Potential impact on weight outcomes.**

- **Has anything happened in the last 12 months, outside of Game of Stones, that might have impacted your food or drink choices or physical activity?**

Prompts: Helpful or unhelpful experiences. If not already discussed:

- Have any health problems had an impact on your weight? (e.g. stress, mental health, physical health problems, family illness)
- Have your circumstances changed in any way that has impacted your weight? (e.g. impact of pandemic, cost of living, employment, relationships)
- **Is there anything that will help you to continue or support your efforts to manage your weight?**

**Prompts:** any strategies that you have found particularly useful? local weight management services, online programmes, apps

**Incentives (SMS+I group only)**

- **What did you think about being offered money for reaching weight loss targets?**

**Prompts:**

How much money did you get? How did this affect your approach to losing weight? Did this change over time?

During the study, the money for incentives was funded by the NIHR, the research arm of the NHS. Who else do you think might be able to provide it in future? Why do you say this?

**Final Questions:**

- **Game of Stones has been designed with men in mind how important was that for you?**

**Prompt:** Is there anyone else you think we should consider offering it to (e.g. some people have suggested it should continue as men only, be available to men with a health condition or on low incomes, be open to women)? Why do you say that?

- **Is there anything about your time on Game of Stones that you’d like to tell me about that I haven’t asked?**

**Thank you for taking part!!**
