# Supplementary material for: ‘They are with me on this journey’, the importance of participant-researcher relationships on attendance at follow-up in a behavioural weight management trial: a qualitative study of participant perspectives
Source: Trials. 2026 Jun 6;27:521. doi: 10.1186/s13063-026-09818-w (PMC13459619; doi:10.1186/s13063-026-09818-w)
Supplement: Supplementary file 5 [file 13063_2026_9818_MOESM5_ESM.docx]

**The Stages of Framework Method**

| **Stage of Analysis** | **Description of Process** |
| --- | --- |
| Transcription | COD and LM transcribed audio-recordings and de-identified transcripts |
| Familiarisation | Researchers (CT. COD, AM) familiarised themselves with the data and became sensitised to early themes by using the audio recordings and transcripts. Analytical memos were created and used throughout for each participant to provide context and reflective notes. |
| Coding | Transcripts were split between researchers involved in the analysis (AM, CT and COD). Open coding was applied to the transcripts labelling short sentences or paragraphs. Although closely related to the questions within the topic guide the codes were inductive. Members of the host trial team also reviewed 2-3 transcripts developing codes for these before an in-person meeting. This allowed discussion of coded text and key themes, issues or discussion points identified from the transcripts. |
| Identifying and developing a thematic framework | CT. AM and COD tested the initial codes on a selection of 8 transcripts. They then met in-person to compare the labels applied and agree on a set of clearly defined codes to apply to all subsequent transcripts. Codes were given a name and labelled with a short description. This formed the working analytical framework which was developed to capture themes arising in the wider host trial as well as specific to the SWAT (See Additional File 5). |
| Applying the analytical framework (Indexing) | Transcripts were divided between CT, COD and AM and coded. Transcripts were uploaded to NVIVO to analysis of the data. This enabled researchers to work separately and then files were brought together to be reviewed and discussed with the wider team. The working analytical framework was systematically applied to all of the study data by indexing all transcripts using codes. Once indexing was complete the early findings and understanding of the data were discussed with the wider project management team. This enabled consideration of information power in relation to answering the research questions. |
| Charting | Framework matrices were utilised in NVIVO and CT, COD and AM mapped and summarised (i.e. charted) all data under each code within the framework. This followed specific conventions to ensure consistency (see Additional File 5) |
| Interpretation | Attributes (e.g. SWAT group) were added to participant rows so that participant characteristics could be considered during interpretation. Comparisons were made regarding SWAT group to explore patterns and clusters within the dataset. One sheet of paper (OSOP) analysis (Ziebland, 2007) was used to map the data clustering similar categories with participant identifiers and describing these together to support interpretation in Microsoft Excel. CT used matrix queries to identify patterns across the data considering these in relation to the research questions. A visual map was developed on the basis of the clusters and identified patterns to organise the categories under themes. |
