# Supplementary material for: ‘They are with me on this journey’, the importance of participant-researcher relationships on attendance at follow-up in a behavioural weight management trial: a qualitative study of participant perspectives
Source: Trials. 2026 Jun 6;27:521. doi: 10.1186/s13063-026-09818-w (PMC13459619; doi:10.1186/s13063-026-09818-w)
Supplement: Supplementary file 6 [file 13063_2026_9818_MOESM6_ESM.docx]

**NVivo Codes (Indexing and Charting)**

1. **Background/participant characteristics**
   1.1. Background information about the participant. *Overview of life circumstances of participant (living circumstances, household composition, house type, employment, hobbies, social life, family life)*
   1.2. Other health problems. *Anything said about* *MLTCs and/or mental health?*
2. **Obesity and overweight**
   2.1. Understanding obesity. *What do they say about men and weight? What do they say about the causes or consequences of obesity generally?*
   2.2. Experiences of obesity and weight management. *What do they say about their attempts to manage their weight, either in the past or present? What prompted them to participate in GoS, for example putting on weight during Covid?*
   2.3. Motivation to lose weight*. What do they say about their motivations to lose weight? Why did they want to lose weight?*
3. **Understandings of GoS**

3.1 Expectations of the trial. *What did they say about what they expected to happen or what they were hoping for? What did they think they were going to get as a result of taking part in the trial?*

3.2. Reflections on trial. *What did they say about recruitment/group allocation?* *Did they suggest any changes that should be made to the trial more generally (not texts/incentives/appointments/website)? What did they say about GoS being designed for men and meeting other men?*

4. **Texts**
   4.1. Delivery. *What did they say about interactivity/two-way communication, what did they say about the timing or duration of texts?*
   4.2. Content. *What did they say about tailoring and personalisation? What did they say about the tone and language of texts?*
   4.3. Suggested changes. *What changes did they suggest in relation to any aspects of the texts?*
5. **Weight targets**
   5.1. Acceptability. *What did they say about how realistic the targets were?*
   5.2. Suggested changes. *What changes did they suggest in relation to any aspects of the weight targets?*
   5.3. General reflections on weight targets. *Did they say anything more general about weight loss targets? Did they say anything about 24m weight loss targets?*
6. **Incentives**
   6.1. General perceptions. *What did they say about the use of incentives in the trial? How did they feel about the incentives? Were incentives generally motivating for them or not? (If linked to specific behaviour change, code to 9.2/9.3)*
   6.2. Suggested changes to incentives. *Did they suggest a different model of incentives? Were there any suggestions about who should pay the incentives going forward? Did they suggest who they thought should be able to receive incentives? (gender, income etc)*
7. **Website**

7.1 Reflections on website. *What did they say about using the website? Did they find the website useful?*

8. **Follow-up appointments**
   8.1. Attending weight assessments. *Any general reflections on what it was like to attend them? Barriers and facilitators to attending? Did their motivation or behaviour change before/after the appointment? What did they say about the practicalities of attending?*
   8.2. Interaction with researcher. *What did they say about the researcher’s approach or manner? What did they say about the value of human contact? What did they say about their relationship with the researcher? What did they say about seeing the same researcher or not?*
   8.3. Suggested changes. *What did they say about how they could be done differently? Any reflections on whether appointments could be done digitally, without contact with a researcher/person?*
9. **Behaviour change**

9.1 Behaviour changes made. *What did they do differently? Did they monitor their weight? Did they count steps? Any unexpected changes in behaviour?*

9.2. Barriers to behaviour change. *What and/or who got in the way of them making changes (e.g. lack of motivation, illness, family changes, incentives)? Do they say anything about their current circumstances that acted as barriers (e.g. cost of living crisis, Covid-related factors, lack of resources in local area)? Anything specific about how texts/incentives/weight targets/follow-up appointments stopped them from changing behaviour?*

9.3 Facilitators to behaviour change. *What and/or who helped them to make changes (e.g. lack of motivation, illness, family changes, incentives)? Did they talk about other groups/organisations/apps that they found helpful? Do they say anything about their current circumstances that acted as facilitators (e.g. Covid-related factors, resources in local area)? Anything specific about how texts/incentives/weight targets/follow-up appointments enabled them to change behaviour?*

9.4. Behaviour changes/strategies for weight maintenance. *What did they say about behaviour changes strategies for maintaining weight loss? (Not continuing weight loss.)*

10. **Outcomes**

10.1 Weight loss. *What did they say about whether or not they lost weight/ meeting the targets set? How do they feel about whether or not they have lost weight?*

10.2. Weight maintenance. *What did they say about maintaining/ sustaining any weight loss as a potential or actual outcome? (Not specific strategies because these go in 9.4)*

10.3. Experiences of GoS. *Anything they said about the general positives and negatives of the trial that aren’t captured elsewhere? Anything they said about being satisfied or dissatisfied? Any upsides or downsides to the trial? (Code neutral responses here too.)*

10.4. Other changes or outcomes*. Did they talk about changes to their wellbeing? Did they talk about any changes or outcomes that they didn’t expect to happen (e.g. to physical health, mental health, or other)?*

11. **Other**
    11.1. Other. *Anything not covered under categories above.*

**Conventions for writing summaries in framework grid**

Please use the following conventions for writing data summaries:

Plain text =direct summary of what participant has said

*Italics =* quote from participant.

**Bold text** = heading (to make summaries easy to read).

Underlined text = a comment, observation or analytical note by the researcher writing the summary.

If a particular sub-theme is not discussed in the interview, please write n/d (not discussed) in the relevant cell.

An extract may be relevant to more than one category. Please index extracts against more than one category as required and cross reference between summaries.
